# Isolated fallopian tube torsion with hydrosalpinx in a 14 year old girl: A case report

**DOI:** 10.1016/j.ijscr.2025.110880

**Published:** 2025-01-13

**Authors:** Dhiran Sivasubramanian, Smrti Aravind, Sathwik Sanil, Virushnee Senthilkumar, V. Rathika

**Affiliations:** a Department of Critical Care Medicine, Christian Medical College, Vellore, India; b Coimbatore Medical College and Hospital, Coimbatore, India; c Institute of Oncology, Sri Ramakrishna Hospital, Coimbatore, India; d Ranga Hospital, Coimbatore, India

**Keywords:** Isolated fallopian tube torsion, Hydrosalpinx, Salpingectomy, Laparoscopic surgery, Case report

## Abstract

**Introduction:**

Isolated fallopian tube torsion (IFTT) is an exceedingly rare but serious cause of acute abdominal pain, especially in pediatric patients, with a reported prevalence of 1 in 1.5 million women. It occurs when the fallopian tube twists around its own axis, leading to venous and lymphatic obstruction, ischemia, and potential necrosis, without involving the ipsilateral ovary. This case report highlights the presentation, diagnosis, and management of IFTT in a 14-year-old girl.

**Case presentation:**

A 14-year-old girl presented with severe right-sided lower abdominal pain and vomiting. Physical examination revealed right iliac fossa tenderness. Imaging, including ultrasonography and magnetic resonance imaging, showed a large right hydrosalpinx. Diagnostic laparoscopy confirmed the presence of isolated fallopian tube torsion with ischemic changes necessitating a right salpingectomy. The postoperative course was uneventful, and she remains well at follow-up.

**Clinical discussion:**

IFTT is rare in children and often confused with appendicitis due to similar symptoms. Diagnosis requires high suspicion and imaging, with ultrasound being the primary modality. Surgical exploration is essential for diagnosis and treatment, with salpingectomy necessary in cases of severe ischemia. In pediatric patients, conservative management with tubal detorsion must be considered in less severe cases.

**Conclusion:**

FTT is a rare but serious condition that can mimic more common abdominal issues. Early recognition and prompt surgical intervention are crucial, particularly in the pediatric population, where concerns about future fertility are more significant. While salpingectomy was performed in this case, further research should focus on assessing the potential benefits of tubal detorsion and revascularization on future fertility outcomes.

## Highlights

• Isolated fallopian tube torsion is a rare cause of acute abdominal pain, especially in pediatric patients.
• IFTT symptoms mimic appendicitis, requiring imaging and surgical exploration for diagnosis.
• Ultrasonography is the best imaging modality, while MRI could also be used.
• salpingectomy is the choice of surgery in ischemic fallopian tube.
• Laparoscopy is gold standard in diagnosis and treatment.

## 1 Introduction

Isolated fallopian tube torsion (IFTT) is an exceptionally rare cause of acute lower abdominal pain, with a reported prevalence of 1 in 1.5 million women [1]. First described by Bland-Sutton in 1890, IFTT involves rotation of the fallopian tube around its longitudinal axis without affecting the ipsilateral ovary, leading to venous and lymphatic obstruction, pelvic congestion, and eventual necrosis of the involved tube [2].

Diagnosing IFTT is particularly challenging due to its nonspecific clinical presentation, which often mimics more common conditions such as appendicitis or pelvic inflammatory disease. This diagnostic uncertainty is compounded in the pediatric and adolescent population, where the condition is even rarer, and premenstrual cases are scarcely reported in the literature. The right fallopian tube is more frequently affected, potentially due to anatomical differences, such as fixation of the left tube by the sigmoid colon or the higher likelihood of imaging the right pelvis for suspected appendicitis [3].

We discuss the case of a 14-year-old girl who presented with severe right-sided lower abdominal pain and vomiting. Subsequent evaluation revealed isolated fallopian tube torsion accompanied by hydrosalpinx—a rare gynecological emergency requiring prompt recognition and laparoscopic surgical intervention. This case highlights the diagnostic challenges and underscores the importance of early laparoscopic evaluation in similar presentations.

## 2 Methods

This work was completed in line with the SCARE criteria [11].

## 3 Case presentation

A 14-year-old girl, presented to the emergency room with sudden onset severe right-sided lower abdominal pain and vomiting for the past 2 days. The pain was not associated with fever, chills, bowel or urinary symptoms, or abnormal vaginal discharge. The patient reported that there were no similar episodes in the past. She had a regular menstrual cycle of 35 days since her menarche 6 months back, with normal menstrual bleeding. The patient has not had sexual intercourse and has no history of previous gynecological or abdominal surgeries.

**Fig. 1:**
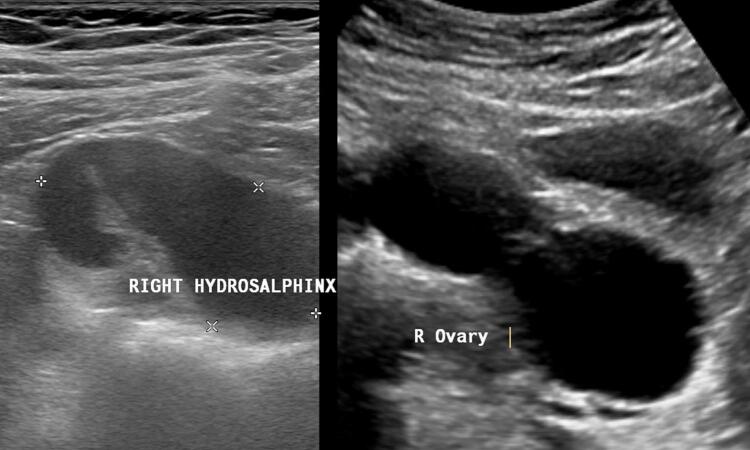
Transabdominal ultrasonography showing large tubular dilated cystic lesion in right adnexa separate from right ovary suggestive of severe right hydrosalpinx.

**Fig. 2:**
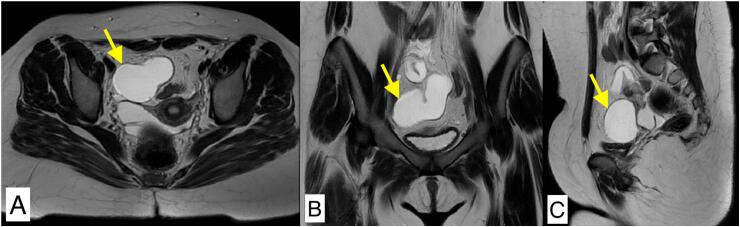
Magnetic resonance imaging (MRI) of pelvis. T2 weighted (A) Axial, (B) Coronal and (C) Sagittal images showing tubular, dilated, convoluted fluid filled structure in right adnexa separate from right ovary.

On physical examination, her vitals were normal, with a blood pressure of 110/70 mmHg, pulse rate 98/min, temperature 98.3oF, and SpO2 99 %. We observed normal development of secondary sexual characteristics in Tanner stages 3–4. Abdominal examination revealed tenderness in the right iliac fossa, with no palpable masses, free of signs of peritoneal irritation, or organomegaly. Bowel sounds were normal. Routine blood investigations showed a hemoglobin of 11.6 g/dl and a mild leukocytosis of 11,300 cells/mm^3^. The urinalysis was normal and the pregnancy test was negative. All other parameters were within normal limits. Transabdominal ultrasonography (USG) showed a normal uterus and normal left and right ovaries. A large tubular dilated cystic lesion of size 8.0 × 3.7 cm was seen in the right adnexa with no flow on Doppler, separate from the right ovary which had flow on Doppler, features suggestive of severe right hydrosalpinx (Fig. 1). No free fluid was observed in the Pouch of Douglas. Magnetic resonance imaging (MRI) showed a dilated, tubular, convoluted lesion in the right adnexa seen separately from the right ovary with predominant fluid content and no internal septation, features again seeming to be suggestive of isolated large right hydrosalpinx (Fig. 2).

**Fig. 3:**
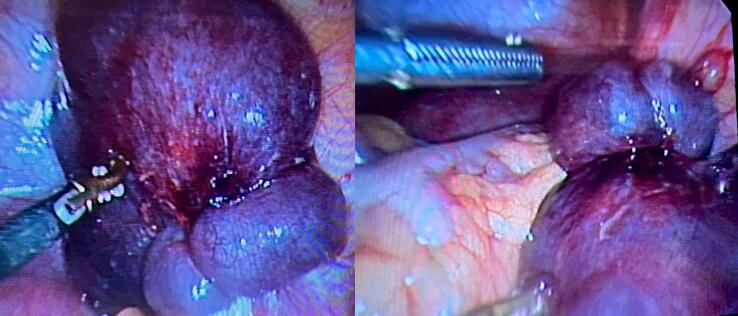
Laparoscopic view of isolated right fallopian tube torsion.

**Fig. 4:**
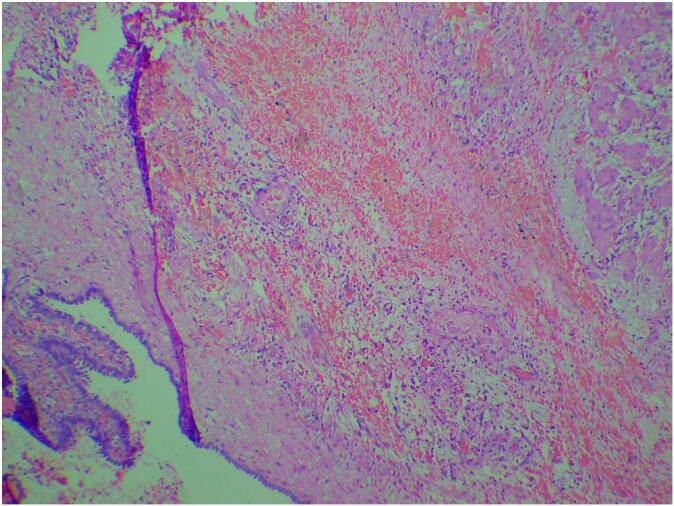
Histopathology of the specimen showing fallopian tube with dilated lumen with flattened plicae. Foci of hemorrhage and hemorrhagic necrosis in the walls.

Given these findings and suspected complications, the patient was hospitalized on the same day. IV antibiotics, IV paracetamol infusion, and IV ondansetron for nausea and vomiting were started. A diagnostic laparoscopy was performed the next day under general anesthesia. During the intervention, we observed that the right fallopian tube was increased in size, with torsion in which the right fallopian tube was twisted 3 times on its own axis and appeared gangrenous (Fig. 3), meanwhile, the right ovary appeared normal with no signs of ischemia. Detorsion of the fallopian tube was attempted, but the tube could not be successfully detorted. In view of signs of severe necrosis of the fallopian tube, a right complete salpingectomy was performed to avoid further complications. The gross surgical specimen measured 10 × 3.5 × 3 cm and later histopathology confirmed the excised structure was a fallopian tube with hemorrhagic necrosis in the walls, without evidence of malignancy (Fig. 4). The postoperative course was uneventful, and she remains well at follow-up.

## 4 Discussion

Isolated fallopian tube torsion (IFTT) is a very rare finding occurring in women of all age groups, it is even rarer in pediatric patients with an estimated prevalence of 0.27 % of all emergency department children with acute abdominal pain complaints [4]. In our literature search, we found a retrospective study by Bertozzi et al. [5] which reported just 20 cases in 26 years at five pediatric surgery units in Italy.

The etiopathogenesis of tubal torsion is not well understood, it involves isolated fallopian tube torsion around the axis formed by the tubo-ovarian ligament. The ovary is typically not compromised due to vascular collateralization provided by the dual blood supply from the ovarian and uterine artery, which forms an arcade around the tube and ovary [6]. The major risk factors associated with IFTT in the adult population are endometriosis, post-surgical adhesions, tubal pathologies such as hydrosalpinx and hematosalpinx, and neoplasms such as ovarian or para-ovarian masses [6,7]. Currently, there are no clearly identified risk factors for IFTT in the pediatric population, with some speculating congenital tubal malformations [8]. Right-sided torsions as seen in our case are more common than left, as the sigmoid colon can act as an obstacle on the left side [7].

Diagnosis of IFTT can be difficult because symptoms are non-specific and can mimic other common conditions. The typical presentation of IFTT is acute lower abdominal pain with nausea and vomiting [3], which could be confused for acute appendicitis, especially in right-sided IFTT like our case. The absence of fever and normal inflammatory marker levels can assist in eliminating the differential diagnosis of appendicitis. Ultrasonography (USG) is the imaging modality of choice because it is easily available, non-invasive, and avoids radiation exposure in pediatric patients [3,7]. Transabdominal USG showing fluid-filled tubular structures (fallopian tubes) folded onto themselves and separated from ovaries is consistent with a diagnosis of hydrosalpinx. Color Doppler may be useful, but the presence of normal flow does not necessarily rule out torsion [3]. Magnetic resonance imaging (MRI) may be used but is less accessible and not very efficient in diagnosing IFTT. Human chorionic gonadotrophin levels can be measured to rule out extrauterine pregnancy. Surgical exploration is the only way to confirm the diagnosis of IFTT [7]. The patient's clinical status will determine the amount of pre-operative checkups and the urgency of surgery [8].

Complete or partial salpingectomy is the therapeutic option in cases of severe ischemic fallopian tubes, where detorsion is not successful, as done in our patient [8]. Conservative surgery with detorsion of the fallopian tube should be attempted initially [7]. In pediatric patients, the future conception capacity of the patient should be taken into account. Therefore, partial tubal salvage and neosalphingostomy should be considered. Salvaging the ovary is a priority in the pediatric population, even if the ovaries appear necrotic [7,8]. The procedure for conservative management after successful detorsion, even in cases of necrosis, was proposed by Boukkaidi et al. They suggested performing a second-look laparoscopy along with salpingoscopy, followed by determining the tubal status using a classification system divided into three grades: I, II, and III. Tubes classified as Grade I and II are considered salvageable and require only salpingoneostomy. In contrast, tubes graded as compromised (Grade III) should undergo salpingectomy. This classification aims to reduce the incidence of recurrence and also the need for salpingectomy [9].

In a multicenter retrospective Italian study conducted by Bertozzi et al., 13 out of 20 patients underwent salpingectomy and also discussed the debatable nature of the surgical management of Isolated fallopian tube torsion and suggested that in the case of a necrotic tube with adhesions and an ischemic tube not showing any revascularisation after detorsion should undergo salpingectomy [5]. As our patient's fallopian tube could not be successfully detorted and showed signs of necrosis, a salpingectomy was required to avoid complications and conservative surgery was not an option.

In terms of the outcomes of conservative vs surgical management, in a systemic review conducted by Kazmi & Gupta et al., 49 patients underwent surgical management showing that most of them were offered salpingectomy while only 4 underwent detorsion alone but later among those who underwent detorsion, 3 had to undergo salpingectomy again due to recurrence. The outcomes of surgical management showed that 55 % were uneventful, 2 % of the patients showed subfertility and the other 29 % went unreported. The patients who were conservatively managed showed comparable outcomes such as among 17 patients 58 % fully recovered while 12 % showed retorsion, and 24 % showed persistence of symptoms [10]. Although there have been incidences of preserving fertility by conserving the fallopian tube, the risk vs benefits must be taken into consideration in patients who show high evidence of necrosis on laparoscopy.

## 5 Conclusion

IFTT remains a rare and challenging diagnosis, particularly in pediatric patients, where its presentation often overlaps with more common conditions like appendicitis. Early recognition through imaging and prompt surgical intervention is critical to prevent complications. Due to the rarity of this condition, case reports and small studies are currently the primary sources of literature. While our patient underwent a salpingectomy, future prospective studies should focus on assessing long-term outcomes of conservative management options, such as detorsion and revascularisation of the fallopian tubes to optimize treatment while preserving fertility, especially in the pediatric population.

## CRediT authorship contribution statement

Dhiran Sivasubramanian - Conceptualization, Writing - Original Draft, Writing - Review & Editing, Project Administration.

Smrti Aravind - Writing - Original Draft, Writing - Review & Editing.

Sathwik Sanil - Writing - Original Draft, Writing - Review & Editing.

Virushnee Senthilkumar - Writing - Original Draft, Writing - Review & Editing.

Rathika V- Writing - Original Draft, Writing - Review & Editing.

## Consent

Written informed consent was obtained from the patient's guardian for publication of this case report and accompanying images. A copy of the written consent is available for review on request.

## Ethical approval

All the data of this study was taken from the medical records of the patient. This report does not contain any personal information that could lead to the identification of the patient. Therefore, it is exempted from ethical approval.

## Guarantor

Dhiran Sivasubramanian

Smrti Aravind

Sathwik Sanil

Virushnee Senthilkumar

Rathika V

## Sources of funding

No funding was provided for the completion of this manuscript.

## Registration of research studies

None.

## Declaration of competing interest

We, the authors of this article, declare that we have no known competing financial interests or personal relationships that could have appeared to influence the work reported in this paper.
